# A transgenic mouse model expressing an ERα folding biosensor reveals the effects of Bisphenol A on estrogen receptor signaling

**DOI:** 10.1038/srep34788

**Published:** 2016-10-10

**Authors:** Thillai V. Sekar, Kira Foygel, Tarik F. Massoud, Sanjiv S. Gambhir, Ramasamy Paulmurugan

**Affiliations:** 1 Departments of Radiology, and Bioengineering, the Bio-X Program, Molecular Imaging Program at Stanford (MIPS), Stanford University School of Medicine, 3155, Porter Drive, Palo Alto, CA 94304-1234, USA

## Abstract

Estrogen receptor-α (ERα) plays an important role in normal and abnormal physiology of the human reproductive system by interacting with the endogenous ligand estradiol (E2). However, other ligands, either analogous or dissimilar to E2, also bind to ERα. This may create unintentional activation of ER signaling in reproductive tissues that can lead to cancer development. We developed a transgenic mouse model that constitutively expresses a firefly luciferase (FLuc) split reporter complementation biosensor (NFLuc-ER-LBD_G521T_-CFLuc) to simultaneously evaluate the dynamics and potency of ligands that bind to ERα. We first validated this model using various ER ligands, including Raloxifene, Diethylstilbestrol, E2, and 4-hydroxytamoxifen, by employing FLuc-based optical bioluminescence imaging of living mice. We then used the model to investigate the carcinogenic property of Bisphenol A (BPA), an environmental estrogen, by long-term exposure at full and half environmental doses. We showed significant carcinogenic effects on female animals while revealing activated downstream ER signaling as measured by bioluminescence imaging. BPA induced tumor-like outgrowths in female transgenic mice, histopathologically confirmed to be neoplastic and epithelial in origin. This transgenic mouse model expressing an ERα folding-biosensor is useful in evaluation of estrogenic ligands and their downstream effects, and in studying environmental estrogen induced carcinogenesis *in vivo*.

Estrogen receptors (ERs: ERα and ERβ) are vital hormone receptors mediating the regulation of cell growth and differentiation in response to estrogens. ERα and ERβ are the two receptors identified to date that dispatch estrogen-mediated signaling in mammalian cells. These two ERs have nearly identical amino acid sequences, approximately 97% similarity in DNA-binding domains, 56% in ligand-binding domains (LBDs), and 24% in their N-terminal ends. The biological action of ERα depends on its constitutively active AF-1 and ligand-dependent AF-2 domains, while ERβ depends only on its AF-2 domain but has a weaker AF-1 domain^1^. ERα and ERβ either homodimerize or heterodimerize upon ligand binding, and then regulate transcription of downstream target genes by interacting with either coactivator or corepressor complexes in the nucleus^2^. A palindromic consensus sequence, termed an estrogen response element (ERE; GGTCAnnnTGACC), has been identified in the regulatory region of many estrogen responsive downstream target genes, through which activated ERs mediate gene expression. However, most of the estrogen target genes including pS2, progesterone receptor and cathepsin D have imperfect palindromic ERE sequences in their DNA^2^,^3^. Activated ERs also bind to other DNA sequences such as cAMP-responsive elements and SP1-binding sites to activate gene expression, and therefore a complex mechanism underlies estrogen-mediated regulation of gene expression^4^,^5^.

ERα has been implicated in several cancer types such as breast, ovarian, colon, prostate, and endometrium^6^,^7^,^8^. For instance, in breast the expression of ER is generally found in ductal and lobular cells but not in stromal epithelial cells^9^, and ERα is strongly over-expressed during the proliferation and invasion of breast cancer. Since ERα mediates the growth and development of several cancers, it has been identified as a major therapeutic target and, therefore, vast arrays of ERα selective ligands have been developed and studied for their potential in controlling the growth of tumor cells by competitively blocking the action of the endogenous ligand estradiol (E2) on ERα^8^,^10^. Ligands selectively bind to the LBD of ERα and regulate the subsequent downstream functions by inducing ER-dimerization. Even a single amino acid change in the LBD of ERα alters the binding affinity of ligands. We previously reported that such a change (ER-LBD-G521T) can completely reduce the binding affinity of E2 while maintaining the normal binding affinity of ER to various other ligands^11^,^12^.

A plethora of ligands has been developed either as agonists or antagonists to ERα, and many of them are currently used in clinical practice for breast cancer treatment and hormone replacement therapy. For example, antagonist ligands are used to treat estrogen responsive breast cancer types, while agonists are used to treat post-menopausal associated complications of hormone replacement therapy^13^,^14^,^15^,^16^,^17^,^18^. Tamoxifen is the first-generation selective ER modulator (SERM) showing antagonist effects in breast, and agonistic ones in uterus and bone. However, breast cancer response to tamoxifen is very successful in initial phases of treatment only, with a significant number of patients developing tamoxifen resistance and tamoxifen induced endometrial cancer^19^. This resistance property has been shown to be associated with variations in the cellular expression level for various downstream effectors of ER-signaling such as coactivators (AIB1) and corepressors (NcoR)^20^. Therefore, there is a real need for alternative ligands that can control the growth of tamoxifen resistant breast cancer.

Many types of ER binding assays have been developed to evaluate the binding affinity of ligands and to screen novel ligands that control the function of ER. *In vitro* autoradiographic and fluorescence polarization based competitive blocking assays are most commonly used to screen ligands, but they do not evaluate the potency of ligands in cells *in vivo* in their native environment where various co-factors are expressed at unique levels. By deleting ERs, ERα^21^, ERβ^22^ and double knockout^23^ mouse models have been generated to study the specific role of these receptors in estrogen mediated signaling. Additionally, transgenic mouse models over-expressing ERα globally in all organs have been generated and used for unraveling the *in vivo* molecular mechanisms of ER activation^24^. However, those transgenic models could not be used to study the role of ERα in specific tissues or organs. To overcome this limitation, mouse models with tetracycline responsive systems were generated and used for studying ERα dominant gain in targeted tissues at particular times of mouse development^25^. Furthermore, to study the ligand-induced downstream effects of ERs^26^ and the *in vivo* dynamics of ERα^27^, transgenic mice expressing luciferase under the control of the estrogen response element (ERE) have also been developed previously. However, at this time there are no transgenic mouse models developed so far to screen ligands that alter the function of ER and simultaneously show the ligand binding affinity as well as its potency.

Bisphenol A (BPA) is an organic synthetic compound in commercial use since 1957 and commonly used to make plastics and epoxy resins. Epoxy resins containing BPA are used in making water bottles, inner linings of water pipes, and various other consumer goods. BPA has been recognized as an endocrine disruptor, as it causes increased body mass index and altered cardiovascular physiology by having agonistic or antagonistic effects on a number of hormone receptors in various small animal species^28^. It was first recognized to possess estrogenic properties as a result of a serendipitous discovery in 1993^29^. Since then, various toxicological studies have revealed that BPA can disrupt endogenous hormones and result in abnormal physiological effects that eventually lead to various diseases. BPA is reported to act as an agonist of ERα and contrastingly as an antagonist of ERβ^30^. Although BPA induces the nuclear transport of ERα and subsequent downstream actions, it is at present considered to be a weak estrogen. BPA has also been shown to act on other important hormone receptors such as the androgen receptor, estrogen-related receptors, and thyroid hormone receptor^28^. Thus, accumulating scientific evidence clearly shows that the safe dose levels of BPA defined by various authorities may not be truly safe for the well being of humans^31^.

Herein, we use a novel transgenic mouse model to study the estrogenic property of this environmental estrogen and its downstream effects in inducing carcinogenesis. We developed mouse harboring fusion genes of split-firefly luciferase (FLuc) and the E2 non-responsive mutant-ERα LBD [NFLuc-ER-LBD(G521T)-CFLuc]. The fusion protein leads to complementation signal upon ligand binding to ER-LBD. We first confirmed the sensitivity and specificity of this transgenic mouse using the well-established ligands Raloxifene (Ral), Diethylstilbestrol (DES), and 4-hydroxytamoxifen (4-OHT) by imaging at different time intervals after systemic and subcutaneous injections. We then evaluated the ER binding affinity and the downstream effects of BPA induced carcinogenesis using continuous long-term bioluminescence imaging of this transgenic mouse model after exposure to environmental doses of BPA.

## Results

### *In vitro* characterization of the plasmid vector expressing NFLuc-ER-LBD(G521T)-CFLuc intramolecular folding complementation biosensor

**Figure 1:**
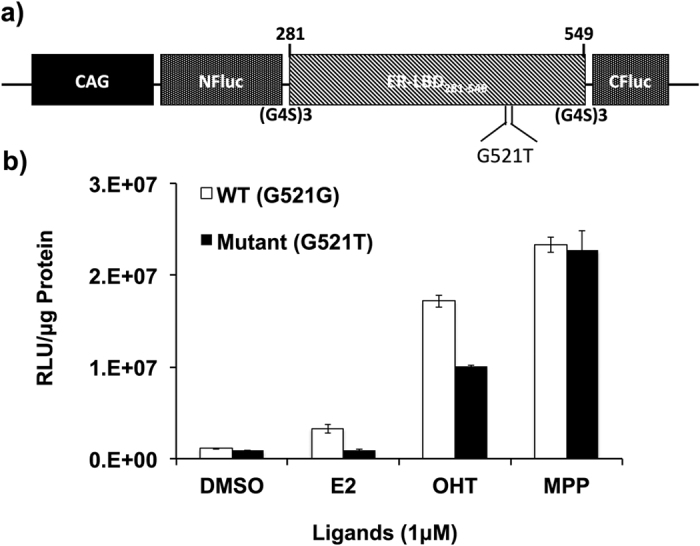
(**a**) Schematic representation of plasmid vector expressing NFLuc-ERα-LBD (G521T)-CFLuc fusion protein under chicken beta actin (CAG) promoter to generate transgenic mice. (**b**) Graph showing normalized FLuc signals measured in 293T cells transfected to express complementation sensors with wild type and mutant ER-LBD [NFLuc-ERα-LBD-CFLuc and NFLuc-ERα-LBD (G521T)-CFLuc] fusion proteins and tested in response to DMSO and different ER-ligands (E2, 4-OHT, and MPP) at 1 μM concentration. WT (G521G) and Mutant (G521T) fusion proteins are labeled in the graph. We previously reported that this mutant (ER-LBD-G521T) can completely reduce the binding affinity of E2 while maintaining the normal binding affinity of ER to various other ligands^11^,^12^. Error bars, mean ± s.e.m.

We constructed a plasmid vector expressing the ligand-induced ER-intramolecular folding biosensor under a chicken-beta actin (CAG) promoter for the generation of transgenic animals. We first tested the sensitivity and specificity of the expressed fusion protein in response to various ER-ligands. HEK293T cells seeded in 12-well plates at 70% confluence were co-transfected with pCAG-NFLuc-ER-LBD-CFLuc (Fig. 1a) and pCMV-RLuc. After 24 h the cells were treated with various ligands for 16 h (Fig. 1b) and subjected to a luminometer assay for RLuc and FLuc activity using coelenterazine and LARII substrates, respectively. Normalized FLuc signals in response to various ligands were recorded and compared relative to normalized FLuc signals measured in DMSO treated cells. Since we used an E2 non-responsive ER-mutant (ER G521T) for vector construction, ligands such as DES, OHT, methyl-piperidinopyrazole (MPP) and Ral induced significantly high intramolecular folding and subsequent FLuc complementation signals, while various E2 analogs (E1, E2, and E3) showed very low levels of FLuc complementation (Fig. 1b). These results clearly demonstrated that the ligand-induced ER-intramolecular folding biosensor expressed under the control of a CAG promoter is specific and sensitive for imaging conformational changes of the ER LBD in response to different ligands in living cells (Fig. 1b).

### Development of transgenic mice

**Figure 2:**
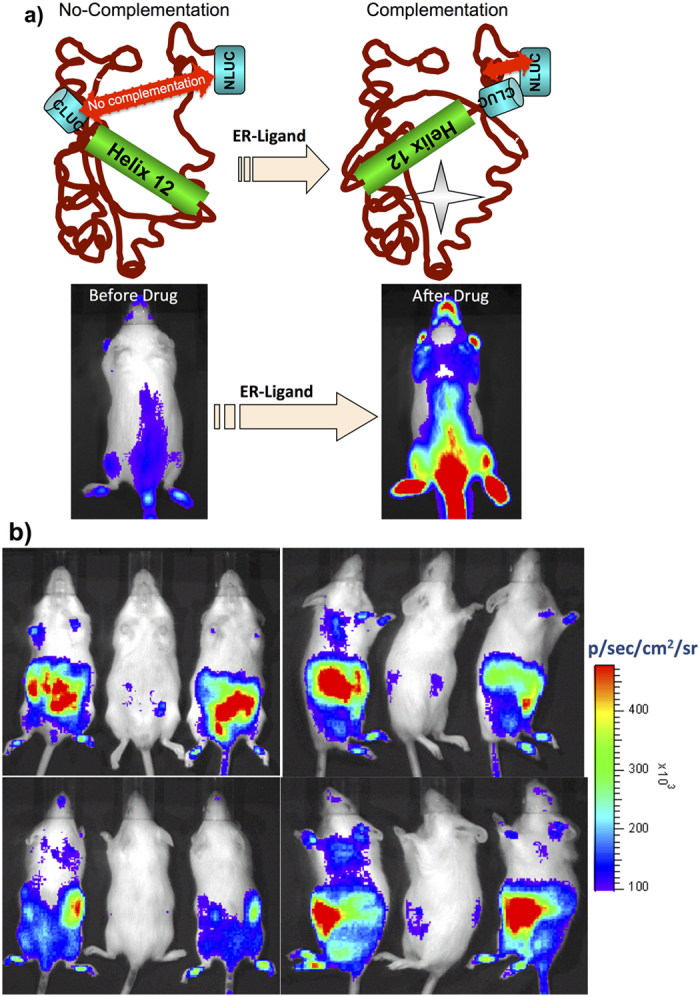
(**a**) Scheme showing the conformational change that occurs in ERα-LBD in response to the binding of ER-ligand (top) and the ensuing FLuc complementation induced by ER, which leads to change in bioluminescence signal in transgenic mice (bottom). (**b**) Screening of transgenic pups for the expression of NFLuc-ERα-LBD(G521T)-CFLuc by optical bioluminescence imaging. Image shows that positive transgenic mice generate FLuc signal when D-luciferin substrate is injected and negative mice yield no signal.

We used the DNA construct expressing NFLuc-ER-LBD (G521T)-CFLuc fusion protein under the Chicken-Actin-CAG promoter (a fusion promoter contain CMV early enhancer with the first exon of chicken beta-actin gene and the splice acceptor of the rabbit beta-globin gene) to create transgenic animals. Transgenic mice produce FLuc signal when binding of ligands to ligand-binding domain ER (ER-LBD) sandwiched in between N- and C- terminal FLuc fragments induce conformational change in the LBD resulting in complementation of FLuc protein (Fig. 2a). We received 32 pups of two litter groups from our transgenic animal facility. We screened the pups using bioluminescence imaging after injecting D-Luciferin, and found six bioluminescence-positive pups. The animals were crossbred and the colony was expanded before we used them for further experiments (Fig. 2a,b).

### Validation of transgenic mice for ER ligand-induced split-FLuc complementation

**Figure 3:**
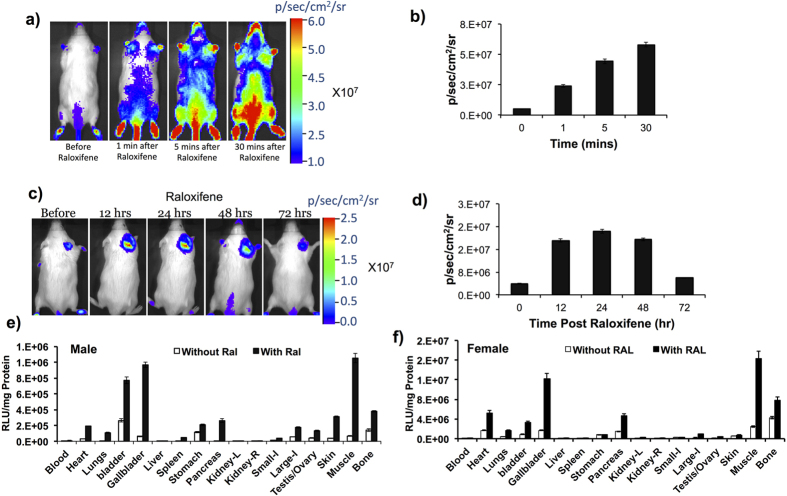
(**a**) Bioluminescence images of transgenic mice expressing NFLuc-ERα-LBD (G521T)-CFLuc before and at different time points (1, 5 and 30 mins) after intravenous injection of 10 μg of Raloxifene. (**b**) Graph showing FLuc signals measured from Raloxifene treated animals in (**a**) over time. (**c**) Bioluminescence images of transgenic animals expressing NFLuc-ERα-LBD (G521T)-CFLuc immediately and at different time points (12, 24, 48 and 72 h) after subcutaneous injection of 1 μg of Raloxifene in 10 μl of sesame oil. (**d**) Graph showing FLuc signals measured from Raloxifene treated animals shown in (**c**). (**e,f**) FLuc signals measured (RLU/mg Protein) in various tissues of male and female transgenic mice in response to the treatment of Raloxifene (10 μg).

The main objective of developing transgenic mice expressing the ligand-induced ER-intramolecular folding biosensor was to screen novel ligands, as well as to study the affinity and potency of ligands that can regulate ER signaling in cells and live animals. Prior to further studies using these transgenic mice, we validated for ligand induced split-FLuc complementation by injecting the ER ligand Ral using two different methods. In the first group Ral was injected intravenously (10 μg in 100 μl PBS) and mice were imaged after also injecting with substrate D-luciferin at subsequent time points (0, 1, 5 and 30 min) using an IVIS spectrum optical CCD camera. The results showed a rapid activation of split-FLuc complementation with an increase in luciferase signal over time (Fig. 3a,b). In a separate experiment, 1 μg of Ral mixed in 10 μl of sesame oil was injected subcutaneously in the shoulder of animals and imaged immediately, and at 12, 24, 48 and 72 h post ligand injection. Ral induced a signal upon FLuc complementation that was significantly higher when compared to vehicle control treated animals, and the signals increased till 48 h post ligand injection, and then diminished at 72 h (Fig. 3b). These results clearly confirmed that the developed transgenic animal showed efficient FLuc complementation, which indirectly reflects upon ER-LBD folding in response to Ral treatment in animals to both modes of deliveries.

### ER ligand specific activation of NFLuc-ER-LBD(G521T)-CFLuc fusion protein expressed in various tissues

To evaluate the ligand-induced FLuc complementation signals in various tissues, we treated the transgenic mice with 10 μg of Ral in 200 μl of PBS by systemic injection, followed by immediate animal euthanasia to excise tissue samples from various organs. FLuc luciferase assay was performed with LARII substrate and the signal was normalized with protein concentration of respective tissue samples (RLU/mg of proteins). FLuc signals were 2–3 fold higher in tissue samples of animals that received Ral when compared to respective tissue samples of vehicle control treated animals. Additionally, ligand-induced signals in tissue samples of female animals were significantly higher (2-fold higher than male) than in tissue samples of male animals (Fig. 3e,f).

### Dynamic Imaging of ER ligand interaction in transgenic mice to evaluate the potency and relative binding affinity of ligands

**Figure 4:**
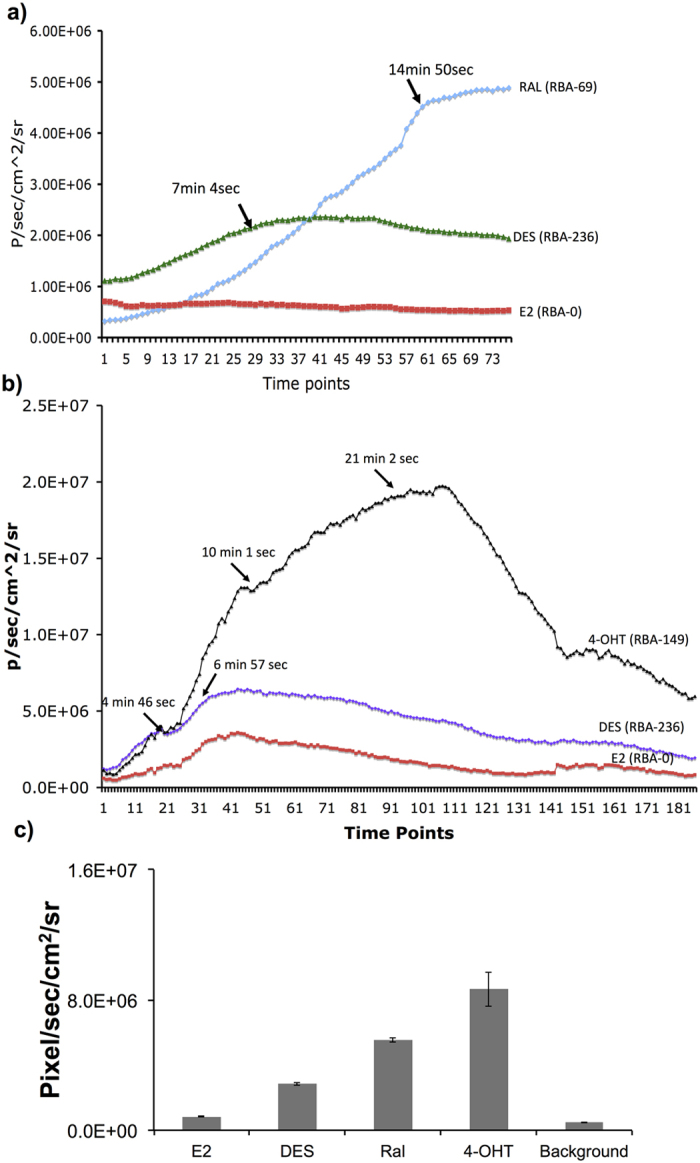
Dynamic imaging of NFLuc-ERα-LBD (G521T)-CFLuc transgenic mice in response to ER-ligands of different binding affinities. (**a**) Graph showing the FLuc signals measured dynamically at different time points after injection with 1 μg of Raloxifene, E2, and DES. (**b**) Graph showing the FLuc signals measured at different time points after injection with 1 μg of E2, DES, and 4-OHT. (**c**) Graph showing maximum FLuc signals measured in transgenic mice injected with ligands E2, Ral, DES and 4-OHT. Error bars, mean ± s.e.m.

After confirming the ligand specific activation of FLuc signal in animals and our capability to image batches of transgenic mice, we evaluated the dynamic imaging ability of the transgenic mice in response to well known ligands such as E2, DES, 4-OHT and Ral to test their potency while comparing with their well established relative binding affinities. We injected transgenic mice with respective ligands (1 μg) and imaged them over time for 30 mins, with 5 sec continuous acquisition in the IVIS Spectrum system, along with continuous infusion of D-Luciferin. Ligand-induced FLuc signals in response to different ligands were plotted over time (Fig. 4a–c; Movies 1,2,3). We drew regions of interest (ROI) for signal detection, estimated the signal, identified the time at which each ligand activates its maximum FLuc signal, and correlated this with their relative binding affinity. The saturation points, i.e., the maximum ligand-induced FLuc signals, varied for different ligands. We observed a saturation point of 15 min and 20 sec for Ral (Fig. 4a,b), whereas DES and 4-OHT reached saturation at about 7 and 10 mins, respectively. These results correlated with the strong relative affinities (DES = 236, and 4-OHT = 149 — for reference, that for E2 and E2-mutant being 100 and 0, respectively) of these ligands for ERα (Fig. 4c). We viewed the dynamic luciferase activation in cine mode, and demonstrated that the target signal appears early after injection if the relative binding affinity of the ligand is strong, whereas the strength of luciferase signal relates to ligand potency (Movies 1,2,3).

### *In vitro* evaluation of primary fibroblasts isolated from transgenic animals expressing split-FLuc complementation sensor (NFLuc-ER-LBD (G521T)-CFLuc) in response to the treatment by various ER ligands and Bisphenol A

**Figure 5:**
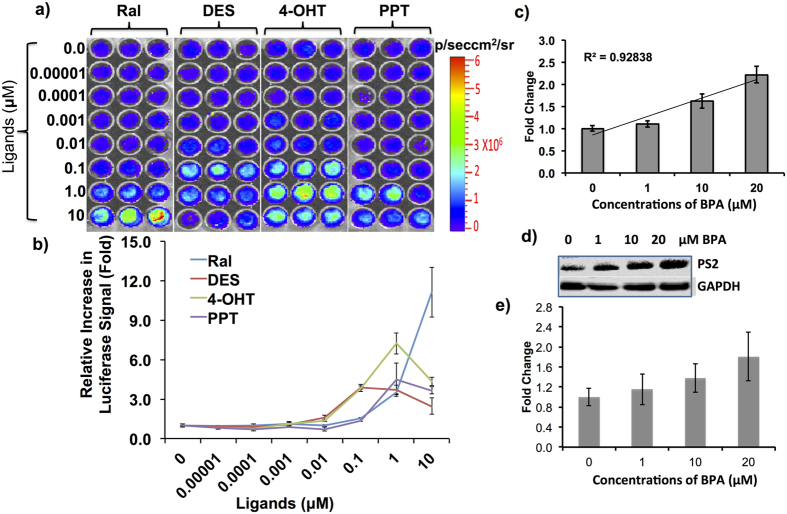
Imaging estrogen, synthetic estrogen, and environmental estrogen (BPA) induced FLuc signals in primary fibroblasts from transgenic mice expressing NFLuc-ERα-LBD (G521T)-CFLuc fusion protein. (**a**) Image showing FLuc signal captured from primary fibroblast cells after treatment with different doses of ligands such as Ral, DES, 4-OHT, and PPT. (**b**) Graph showing the fold-change in FLuc signals measured in (**a**). (**c**) Graph showing FLuc signals measured from the fibroblast cells after treatment with different concentrations of BPA. (**d**) Immunoblot analysis showing estrogen responsive pS2 protein levels in primary fibroblasts isolated from transgenic mice in response to treatment with different doses of BPA (0–20 μM). (**e**) Graph showing the fold change of pS2 protein level normalized to GAPDH shown in (**d**). Error bars, mean ± s.e.m.

Previous reports have identified BPA as an xenoestrogen that can cause pre-neoplastic and neoplastic transformation in rat mammary glands. Since we planned to evaluate our transgenic animals for BPA-induced estrogenic property and its associated toxicity, we first tested fibroblast cells isolated from these transgenic animals for the presence of sensor response to various ER-ligands, including BPA. We isolated fibroblasts and maintained them in DMEM medium. Since the transgenic mice were already validated by bioluminescence imaging using ligands such as Ral, DES and 4-OHT, to further confirm the behavior of primary fibroblasts, and to study the dynamic range of ligands, we treated primary fibroblast plated in 96-well plates (10,000 cells/well) with various doses (0 to 10 μM) of ligands such as Ral, DES, 4-OHT, and Propyl Pyrazole Triol (PPT) and measured FLuc signals 12 h post incubation. Similarly, the cells seeded in 12-well plates at 70% confluence (100,000 cells/well) were incubated with various doses of BPA (0–20 μM) for 12 h before assessing them for luciferase activity. All four ligands (Ral, DES, 4-OHT, and PPT) showed dose dependent FLuc complementation, with the higher fold changes of FLuc signals at different doses that matched the relative binding affinity of all four ligands. Ral, DES, 4-OHT, and PPT, respectively, showed a fold changes of 11.1 (10 μM), 3.7 (0.1 μM), 7.24 (1 μM), and 4.48 (1 μM) concentrations when compared to control (Fig. 5a,b). Since BPA is a low affinity xenoestrogen we used 0–20 μM concentrations for treatment. The results clearly showed a significant level of BPA-induced conformational changes in ER-LBD as a measure of FLuc complementation (2.2-fold at 20 μM concentration, Fig. 5c). Further, the dose study analysis showed significant correlation of the concentrations of BPA used in the study with the FLuc complementation signal (R^2^ = 0.92) (Fig. 5c). To further scrutinize this observation, we performed an immunoblot analysis with primary fibroblast lysates exposed to different concentrations of BPA (0–20 μM) to measure the downstream ER target protein pS2 levels. Immunobloting showed linearly increasing concentrations of pS2 levels in response to increasing concentrations of BPA, confirming the BPA mediated transactivation of ERα (Fig. 5d,e).

### Imaging Bisphenol A activated ER intramolecular folding changes in transgenic mice expressing NFLuc-ER-LBD (G521T)-CFLuc fusion protein

**Figure 6:**
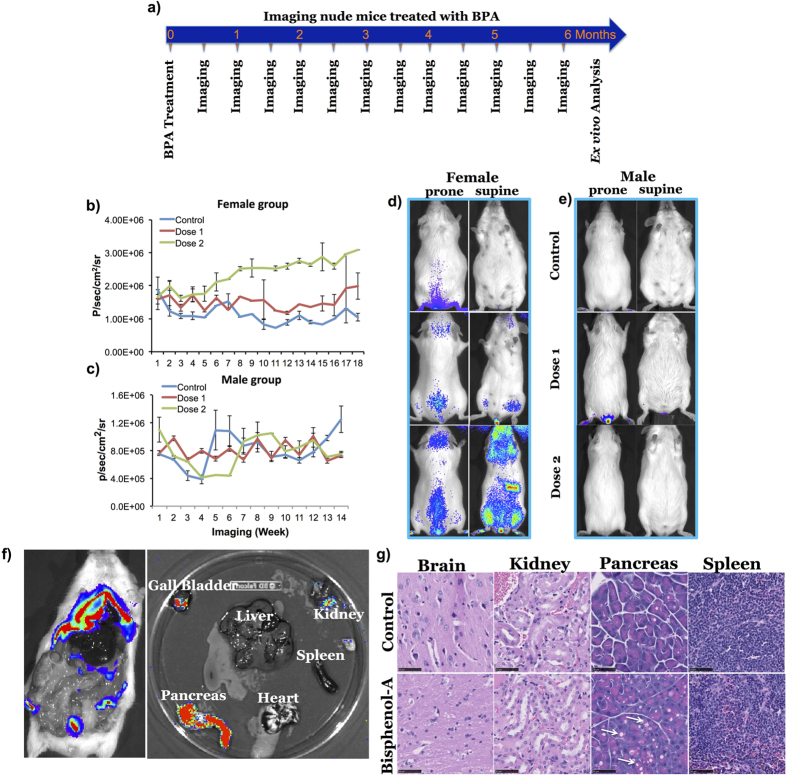
*In vivo* imaging of transgenic mice expressing NFLuc-ERα-LBD (G521T)-CFLuc fusion protein in response to the treatment of BPA. (**a**) Scheme showing the timeline of treatment and *in vivo* imaging schedule for transgenic mice in response to BPA treatment. (**b,c**) Graph showing FLuc signals measured in female and male transgenic mice after long term exposure to two different doses (2.8 and 5.6 nM) of BPA in water for six months. (**d,e**) Figure showing bioluminescent images of female (**d**) and male (**e**) transgenic mice exposed to 2.8 and 5.6 nM BPA. (**f**) Bioluminescent images of pancreas, heart, spleen, kidney, liver, and gallbladder of female transgenic mice exposed to 5.6 nM BPA at the end of the treatment period. (**g**) Figure showing microscopic images of H&E stained brain, kidney, pancreas, and spleen slices of representative female transgenic mice from the BPA treatment group. The tissue slices are from a female animal treated with 5.6 nM dose BPA for six months. Error bars, mean ± s.e.m.

Next, we studied the ER activation by BPA. BPA at concentrations of 2.8 and 5.6 nM (latter value reported as contaminant threshold in the environment by Kang *et al*.)^32^ were given to mice in drinking water for a period of 6 months and imaging was performed fortnightly during the treatment period for tissue specific activation and luciferase complementation signal (Table 1). FLuc signal recorded from female transgenic mice was significantly high after a 5.6 nM dose of BPA (3.09 × 10^6^ ± 2.75 × 10^4^ p/sec/cm^2^/sr), which was 3-fold higher when compared to the vehicle treated transgenic mice group (1.04 × 10^6^ ± 1.12 × 10^5^ p/sec/cm^2^/sr) (Fig. 6b–e). *Ex vivo* imaging of vital organs clearly showed a high BPA-induced FLuc signal in the pancreas, moderate signal in the kidneys, and no significant FLuc signal observed in organs such as liver, heart and spleen (Fig. 6f). Histological examination showed pronounced vacuolation in the pancreas of BPA treated animals when compared to organs from vehicle treated animals (Fig. 6g).

### Bisphenol A induced tumor-like outgrowths in transgenic mice expressing NFLuc-ER-LBD (G521T)-CFLuc fusion protein

**Figure 7:**
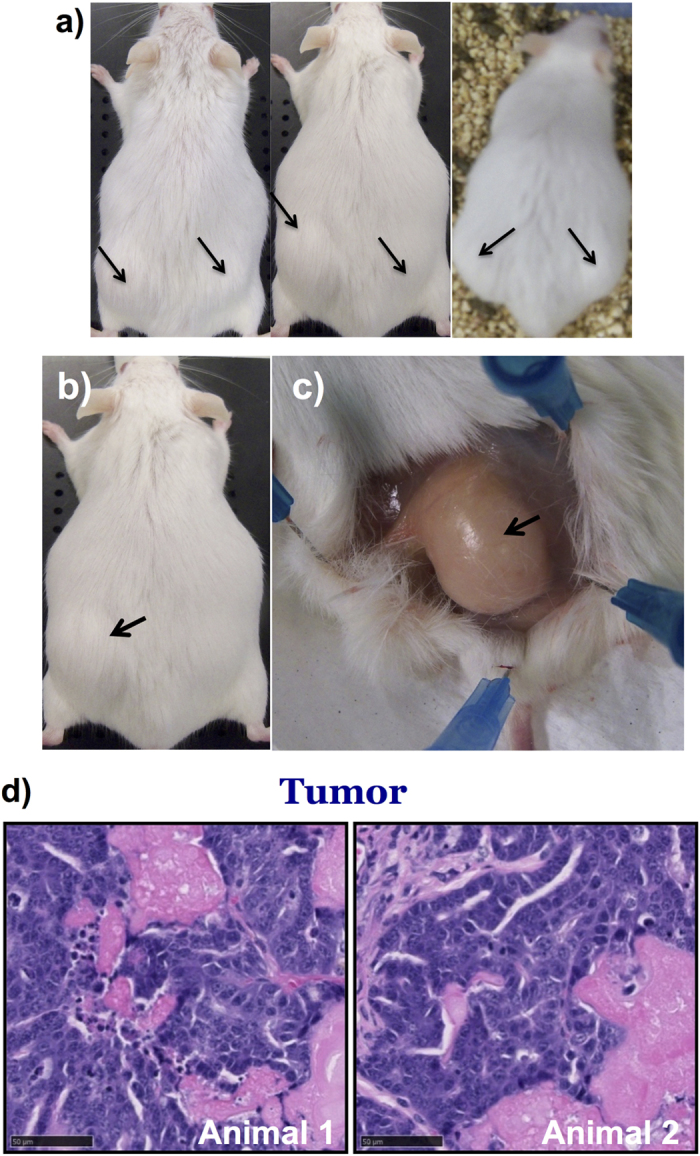
Neoplastic outgrowths in female transgenic mice treated with BPA. (**a**) Photographs of representative female transgenic mice that developed neoplastic outgrowths after treatment using 5.6 nM BPA. (**b,c**) Images showing the neoplastic outgrowth in flank muscles of a female transgenic mouse exposed to 5.6 nM of BPA for six months. (**d**) Microscopic images of H&E stained neoplastic outgrowths from two different female transgenic mice with histologically confirmed adenocarcinoma.

In the course of treating mice with BPA, we observed tumor-like outgrowths in the flanks of transgenic animals treated with 5.6 nM BPA (Fig. 7a). Interestingly, only female transgenic mice exhibited the outgrowths which correlated with the upregulated activation of BPA-induced FLuc complementation signal. BPA-induced tumor growth in the flanks was observed in about 40% of female transgenic mice (6 of 15 animals), and only in females older than 6 months receiving higher BPA doses. None of the male transgenic mice treated with similar doses exhibited any tumor growth. Upon surgical exposure, the tumor-like growths appeared on the surface of muscle just beneath the skin (Fig. 7b). We entirely speculate that the characteristic location of these tumors, i.e., being morphologically related to muscle surface, perhaps was owing to our use of the chicken beta actin promoter to drive expression of our transgene. Even though the overall luciferase signal was activated in these animals, the FLuc signals in these tumors was not significantly different compared to other groups that showed no tumor growth (i.e., females receiving lower doses, and males receiving low and high doses). We excised a portion of a tumor and examined this histologically by H&E staining. This, as well as a formal pathology analysis, revealed the epithelial origin of the neoplastic outgrowth as adenocarcinoma (Fig. 7c).

## Discussion

Estrogen receptors control the development and proliferation of various hormone responsive tumors such as breast, uterine and endometrial. As such, ER has been identified as a potential therapeutic target to curtail the proliferation and spread of hormone responsive cancers^33^. An array of ER ligands with various properties have been evaluated, and many of these are used clinically^34^. Different types of ligands have also contributed significantly to the study of the biology of ERs and to understanding the signaling mechanisms that determine various stages of hormone responsive cancers^35^. E2 is an endogenous hormone that governs the proliferation of cells in various tissues, and has been implicated in the onset of various malignancies such as breast and ovarian cancers^36^. To combat the action of E2 in cancer onset and proliferation, various synthetic ligands have been identified. Ligands such as 4-OHT are SERMs that antagonize the action of E2 in mammary cells and also can act as agonists in other tissues, while ligands such as ICI 182,780 and its derivatives are considered selective ER degraders (SERDs) that act as antagonists to E2 in all tissue types^37^. In addition to conferring agonistic and antagonistic properties, ligands in general reduce the transcription of ER target genes^38^. Although ligands such as tamoxifen are recognized as important therapeutic agents to help contain various ERα positive cancer types, resistance against tamoxifen is a major concern in the clinical setting, and no effective countermeasures for this are available to date^39^. Potential alternative therapeutic ligands could offer a solution to this significant limitation.

Screening for promising therapeutic ligands that could replace tamoxifen and thereby avoid the drawback of tamoxifen resistance would be highly desirable. The screening of such ligands could be accelerated with use of suitable assays that mimic the *in vivo* conditions that exist in mammalian cells. Currently, new ER ligands are evaluated by *in vitro* methods such as competitive blocking assays that evaluate their relative binding affinities. However, assessment of potential ER ligands that confer promising therapeutic effects *in vivo* within cells, in small animals, and also in humans would not be possible with such assays. To address this issue, we developed a transgenic mouse model expressing an ER-LBD mediated split FLuc complementation biosensor. The ligand screening capabilities and sensitivity of the developed transgenic model were first evaluated using well-known ligands such as E2, DES, OHT, MPP and Ral. We found that ligand binding mediated FLuc signals obtained during dynamic studies of Ral, OHT, and DES were in agreement with the reported relative binding affinities for the respective ligands. The site-specific mutation of ER-LBD (G521T) in this transgenic mouse model prevents the binding of endogenous ligand E2, and therefore facilitates the screening of novel potential exogenous ligands. Upon inspection of the ligand-binding pocket surrounding E2 using a space filling model, we found that this particular glycine residue located at amino acid position 521 (G521) is in close proximity to carbon 15 and 16, which loom above the edge of the D-ring of the steroidal core of E2. In this model we also substituted threonine at position 521 and found that the larger residue clashes sterically with both the D-ring atoms and even with the angular methyl group. Moreover, this transgenic mouse provides additional useful information regarding ligand potency, based on the level of complementation induced by each ligand. This may promote the rapid *in vivo* identification of new ligands possessing different characteristics such as those that may be SERMs, SERDs, or partial ER agonists/antagonists.

In addition to thoroughly characterizing the transgenic animals for their potential in drug screening, we also used this model to study the ER signaling mediated neoplastic potential of BPA. Continuous exposure to BPA for six months significantly induced ER activation in female transgenic animals when compared to male animals that received similar doses of BPA in drinking water. Additionally, BPA induced tumor-like outgrowths in the flank regions of approximately 40% of female transgenic mice that received 5.6 nM of BPA (equal to the current environmental contamination level). Interestingly, we observed that animals older than 6 months of age were highly prone to developing neoplastic growths when compared to younger mice that were less than 5 months old. BPA had already been shown to have mild estrogenic activities, and was therefore implicated in carcinogenesis of hormone responsive cancers^40^,^41^. It has also been shown that BPA at environmental concentrations could cause uterine sarcoma, and mammary adenocarcinoma in mice^42^. We found tumor growth mainly in the muscles of female mice. The possible explanation for the rapid development of tumor outgrowth in muscles compared to other organs may be the presence of stromal cell populations in muscles. Conceivably, another reason could be that any possible tumors appearing in other internal organs may be much smaller and therefore not detectable at the time we found large tumor masses in muscles. Future studies with continuous maintenance of animals for extended periods after surgically removing the primary tumor in muscle can possibly further elucidate the temporal aspects and selective locations of carcinogenesis and subsequent tumor growth, as well as explain the role of BPA in various other organs. Although the underlying molecular mechanism of BPA carcinogenesis has not been completely demonstrated, several previous reports revealed its role in altering major cell signaling pathways and regulating the expression of proteins that controls cell cycle and apoptosis^43^,^44^,^45^, besides its interactions with major hormone receptors such as ERα, ERβ, and AR. BPA treatment has also been shown to induce the expression of microRNAs in placental cells^45^. Additionally, BPA alters the histone methylation status of H3K27 methylation marks in chromatin by regulating the expression of EZH2, a histone-lysine N-methyltransferase enzyme^46^. Recently it was also shown that BPA could induce human prostate stem-progenitor cells and favors prostate specific carcinogenesis. However, for the first time, we directly relate long-term exposure to BPA at its environmental equivalent dose to its carcinogenic effect in experimental animals. Specifically, our results indicate that long-term exposure to BPA can induce carcinogenic effects with a concomitant increase in luciferase signal in the transgenic animals. This animal model also offers the potential to simultaneously evaluate ligand potency and biological characteristics, opening new avenues to discover novel ligands that can replace tamoxifen to treat subtypes of tamoxifen-resistant breast cancer.

## Conclusion

We developed and validated a transgenic mouse model expressing a split FLuc complementation biosensor (NFLuc-ER-LBD_G521T_-CFLuc), to help screen novel ligands that bind to ERα, thus activating or repressing its downstream reactions. Applications of this transgenic mouse model confirm the estrogenic properties of established ligands such as Ral, DHS, E2, and 4-OHT, as well as the weakly estrogenic BPA. Long-term exposure to BPA was found to induce tumor-like outgrowths in female transgenic mice, confirmed to be epithelial neoplasms. This preclinical transgenic model holds significant promise for screening potential small molecules acting as agonists or antagonists of ERα, and therefore could expedite the development of alternative therapeutic agents to treat tamoxifen-resistant breast cancer.

## Materials and Methods

### Chemicals, Enzymes, and Reagents

Cell culture plates, FBS, penicillin, streptomycin, sodium bicarbonate, and cell culture medium were purchased from GIBCO BRL (Frederick, MD). Lipofectamine 2000 transfection reagent and pcDNA3.1 (+) eukaryotic expression vector for constructing different plasmid vectors used in this study were purchased from Invitrogen (Carlsbad, CA). Ampicillin for bacterial culture and dimethyl sulfoxide (DMSO) were purchased from Sigma (St. Louis, MO). Bacterial culture media were purchased from BD Diagnostic Systems (Sparks, MD). All restriction, modification, and T4-DNA ligase enzymes were purchased from New England Biolabs (Beverly, MA). The plasmid vector encoding E2 non-responsive ER-LBD split-complementation fusion protein was constructed using various DNA fragments available from our plasmid bank. LARII substrate for firefly luciferase assay and 5X passive lysis buffer was purchased from Promega Corp (Madison, WI). The plasmid extraction kit and DNA gel elution kit were purchased from Qiagen (Valencia, CA) and Epoch life sciences (Missouri city, TX). D-luciferin was purchased from (Biosynth, Switzerland) and coelenterazine was purchased from Nanolight (Pinetop, AZ). Polymerase for PCR amplification was purchased from 5 Prime (Gaithersburg, MD). Estrogen inducible pS2 antibody was purchased from Abcam (Cambridge, MA). Primers for PCR amplifications were synthesized by Stanford Protein and Nucleic Acid (PAN) facility. Sequencing of plasmid vectors were carried out by Sequetech DNA sequencing service (Mountain View, CA). Site-directed mutagenesis was performed using the Stratagene kit (La Jolla, CA). Ligands such as E2, Ral, 4-OHT, DES, and PPT were purchased from Sigma (St. Louis, MO). Collagenase Type I enzyme and Bisphenol A were purchased from Sigma (St. Louis, MO).

### Construction of Plasmids

The plasmid for transgenic mice development was constructed by inserting PCR amplified ER-LBD_G521G_ domain in between NH_2_-terminal (NLUC, amino acids 1–398) and COOH-terminal portion (CLUC, amino acids 394–550) of the Fluc gene in pCDNA3.1(+) vector (Invitrogen, Carlsbad, CA) modified to express the puromycin resistance gene. The CMV promoter of pcDNA (3.1) was further modified with chicken β-actin promoter (CAG) to drive the expression of NFLuc-ER-LBD-CFLuc fusion protein. After the construction of plasmid expressing NFLuc-ER-LBD-CFLuc fusion protein, G521T mutation was created in ER-LBD (ER-LBD_G521T_) by using the Stratagene site-directed mutagenesis kit.

### Cell transfection and luciferase assay

HEK293T cells were transfected with plasmid vector expressing NFLuc-ER-LBD_G521G_-CFLuc and NFLuc-ER-LBD_G521T_-CFLuc fusion proteins by using Lipofectamine 2000 transfection reagent from Invitrogen (Carlsbad, CA), according to the manufacturer’s protocol. In brief, the cells were transfected with 0.5 μg of DNA and 1.5 μl of Lipofectamine 2000 per well (1:3 ratio) in 12-well culture plates. Plasmid expressing renilla luciferase (RLuc) was co-transfected at every transfection to normalize the transfection. Luciferase assay was performed after 16 h of exposure to various ER-ligands. Cell pellets were lysed in 200 μl of 1X passive lysis buffer and centrifuged at 10,000 g for 5 min at 4 °C to remove cell debris. Clear cell lysate was transferred to a fresh microfuge tube, and 10 μl of lysate were mixed with 1 μg of substrate in 100 μl PBS (Coelenterazine for RLuc activity) and 50 μl of LARII for FLuc activity, just before recording the signal in luminometer.

### Generation of Transgenic Mouse

The plasmid vector (pCAG-NFLuc-ER-LBD_G521T_-CFLuc) used for the generation of the transgenic mouse model was developed as described above. The complete 4.5 kb fragment of fusion reporter gene with the promoter and the PolyA-tail (CAG-NFLuc-ER-LBD-CFLuc-PolA) was released from pcDNA-CAG-NFLuc-ER-LBD-CFLuc vector by *SalI* and *XmnI* double digestion, and gel purified at a concentration of 50 ng/μl. The pCAGGS vector was earlier modified by addition of suitable restriction enzyme sites through a small nucleotide linker for convenient release of the fragment without any additional sequence. The ER-transgenic mouse line was made using FVB background. The transgenic was made by pronuclear injection of CAG-NFLuc-ER-LBD-CFLuc-PolA reporter gene fragment with the help of the transgenic animal facility at Stanford University. Injected eggs were re-implanted by oviduct transfer into outbred pseudopregnant females generated by mating to vasectomized males. After the microinjections and oviduct transfers, mice were housed in the facility until pups were weaned at 21 days after birth. Pups were screened by *in vivo* BLI by i.p. injection of 25 μl of 30 mg/ml D-Luciferin. We bred all the positive transgenic mice for two generations, and then used them for various experiments as described in this manuscript.

### Isolation of Primary Fibroblasts

Primary fibroblast cells were isolated from adipose tissues of transgenic female mice. Briefly, adipose tissue samples were collected from euthanized female mice and transferred immediately to 15 ml falcon tube containing sterile 25 mM HEPES buffer (pH 7.4). All surgical procedures were performed with sterile tools. Adipose tissue samples were chopped into small pieces and transferred to 5 ml of collagenase type I (1 mg/ml) dissolved in HEPES buffer and incubated at 37 °C for 20 min with intermittent mixing with a sterile Pasteur pipette. Tissue suspension was observed under microscope at regular intervals to ensure complete dissociation of adipose tissue. Tissue suspension was filtered through a 20-μM-nylon filter and centrifuged for 3 min at 300 g, and the pellet was washed three times with sterile HEPES buffer. For culturing primary fibroblast cells, a cell pellet was suspended in sterile DMEM medium containing 10% FBS and 1% penicillin-streptomycin and maintained following regular cell culture conditions.

### Bioluminescence imaging in living mice

All animal experimental protocols were approved by Stanford University Administrative Panel on Laboratory Animal Care (APLAC) and research committee. All methods were carried out in accordance with protocol approved by the Institution (#APLAC-27980). Mice were anesthetized using isoflurane (2% isoflurane in 100% oxygen, 1 liter/min) during all ligand injection and imaging procedures. Imaging was done using a cooled charge-coupled device camera (IVIS-spectrum, Perkin Elmer, Waltham, MA). The animals were imaged just before and after ligand injections, and during various time points after BPA treatment. We also performed imaging of animals at different time intervals for dynamic imaging of ligands. BPA-treated animals were imaged fortnightly for a period of six months. D-Luciferin (3 mg in PBS) was injected i.p, and animals were imaged with the IVIS imaging system (IVIS-spectrum; Perkin Elmer, Waltham, MA) with an acquisition time of 1 min each until the signal reached the peak. For *ex vivo* imaging, organs from experimental animals were excised immediately after they were euthanized and placed in a 10 cm petri dish and imaged. Images were obtained and analyzed using Living Image Software. To quantify the measured light, regions of interest were drawn over the area of signal emission or whole animal in BPA treatment, and the maximum photons/sec/cm^2^/steradian (sr) was obtained, as validated previously.

### Ligand-mediated activation of NFLuc-ER-LBD(G521T)-CFLuc fusion protein in tissues

Transgenic mice were euthanized immediately after Raloxifene (10 μg in 200 μl of PBS) injection and tissue samples from various organs were excised. Tissue samples were lysed with passive lysis buffer (Promega, Madison, WI) and centrifuged at 10,000 g for 5 min to remove cell debris. To perform the luciferase assay, 10 μl of supernatant was added to 100 μl LARII substrate just before reading in the luminometer. Protein concentration of every tissue lysate was measured by Bio-rad assay and FLuc signal was normalized with protein concentration of respective tissue samples (RLU/mg of proteins).

### Bioluminescence imaging in mice to dynamically measure ER-ligand activation of intramolecular folding sensor

The transgenic animal was used for imaging to study the dynamic activation of ER-ligand induced intramolecular folding assisted complementation using different imaging conditions. In this set-up, the animals were placed with a catheter in the tail vein to continuously infuse D-Luciferin to maintain the concentration of circulating D-Luciferin at a constant level. Mice were anesthetized using isoflurane (2% isoflurane in 100% oxygen, 1 liter/min) during the entire imaging procedures. Imaging was performed using a cooled charge-coupled device camera (IVIS-spectrum; Perkin Elmer, Waltham, MA). D-Luciferin at 5 mg/mL in PBS was used for injection. The substrate was administered at a constant rate of 65 μL/min using an automated infusion pump (GeniePlus, Kent Scientific, Torrington, CT) to achieve steady state. After initial infusion for 5 mins to pre-saturate the animal, 1 μg of ligand in 10 μl volume PBS was injected subcutaneously and the images were dynamically acquired every 5 seconds for 30 mins. The ROI was drawn to quantify the luciferase signal and evaluated for the potency of the ligand based on the intensity of signal and relative binding affinity based on the time of peak activation.

### BPA long term study protocol and conditions and care while handling carcinogen

Ten- to 12-week old transgenic mice were randomly divided into male and female groups comprising 10–15 animals in each group. Animals were housed in disposable cages and drinking water was provided with BPA-free 100 ml bottles following the Stanford University chemical hygiene plan for animal research. One of the three groups was maintained for control animals by giving drug-free water, while the other two groups were given 2.8 and 5.6 nM of BPA, respectively, throughout the experimental period of 6 months. Transgenic animals in the experimental groups were imaged every two weeks for a maximum period of 6 months (Fig. 6a). Bioluminescent signals were captured for 2 minutes in an IVIS spectrum-imaging instrument by administering D-luciferin substrate through the tail vein by intravenous injection. After 6 months, transgenic animals were euthanized and bioluminescent signals were captured from internal organs such as liver, pancreas, heart, kidney, spleen, and gall bladder. Tissue samples of organs were fixed and used for histological analysis.

### Immunohistochemistry

At the end of BPA treatment, transgenic animals were euthanized and specific organ samples were fixed in 4% paraformaldehyde and used for paraffin embedded cutting of slices for H&E staining. Animals that developed tumor-like outgrowths were sacrificed and portions of tissues were fixed in formalin and in OCT. Formalin-fixed paraffin-embedded tumor xenografts were processed for histological analysis by H&E staining for necrosis scoring in tumors.

### Immunoblot Analysis

For immunoblot analysis, mouse fibroblast cells were washed with PBS after treating them with various concentrations of BPA to remove any traces of culture medium and debris, and lysed in RIPA buffer (Pierce Biotechnology, Rockford, IL, USA). The lysate was sonicated thoroughly to ensure the complete dissociation of proteins. The lysate was then centrifuged at 16,000 g for 15 min at 4 °C, and the protein content of the supernatant solution was estimated by a Bradford protein assay kit (Bio-Rad, USA). A total of 30 μg of protein was resolved in 4–12% gradient SDS/PAGE (Invitrogen) and electroblotted onto a 0.2 μM pore size nitrocellulose membrane (Schleicher & Schuell Biosciences, GmbH). Pre-stained protein marker was used at every run to confirm the molecular mass and complete transfer of protein to the membrane. The membrane was blocked in 5% non-fat dry milk in TBS-T (TBS with 0.05% Tween 20) buffer for 1 h. The membrane was further incubated in the same blocking solution with pS2 specific antibody overnight at 4 °C on a rotating platform. The membrane was washed 3 times with TBS-T and incubated with HRP-conjugated goat anti-rabbit antibody for 1 h at room temperature. The membrane was washed 3 times with TBS-T buffer before incubation with the ECL chemiluminescent HRP substrate (Life technologies, Grand Island, NY), following the manufacturer’s instructions. The same membrane was stripped and re-probed with mouse anti-human GAPDH antibody (Sigma, St. Louis, MO) to control for protein loading. The signal was detected using IVIS optical CCD camera imaging. Drawing ROI over the bands quantitated the signal.

## Additional Information

**How to cite this article**: Sekar, T. V. *et al*. A transgenic mouse model expressing an ERα folding biosensor reveals the effects of Bisphenol A on estrogen receptor signaling. *Sci. Rep.* **6**, 34788; doi: 10.1038/srep34788 (2016).

## Supplementary Material

Supplementary Information

Supplementary Movie 1

Supplementary Movie 2

Supplementary Movie 3

Supplementary Movie 4

## Figures and Tables

**Table 1:** Treatment conditions, Imaging, and number of animals used for BPA treatment.

	Female			Male		
	Control	BPA- Dose 1	BPA- Dose 2	Control	BPA- Dose 1	BPA- Dose 2
No. of Animals	N = 10	N = 15	N = 15	N = 5	N = 5	N = 10
Duration of Exposure	6 months	6 months	6 months	6 months	6 months	6 months
Imaging	Fortnight	Fortnight	Fortnight	Fortnight	Fortnight	Fortnight
Tumor Growth	No	No	N = 6	No	No	No
