# Supplementary material for: A transgenic mouse model expressing an ERα folding biosensor reveals the effects of Bisphenol A on estrogen receptor signaling
Source: Sci Rep. 2016 Oct 10;6:34788. doi: 10.1038/srep34788 (PMC5056407; doi:10.1038/srep34788)
Supplement: Supplementary Information [file srep34788-s1.doc]

**A transgenic mouse model expressing an ERα folding biosensor reveals the effects of Bisphenol A on estrogen receptor signaling**

Thillai V Sekar, Kira Foygel, Tarik F Massoud, Sanjiv S Gambhir* and Ramasamy Paulmurugan*

**Movie Legends:**

**Movie 1**. Video showing the real-time luminescence signal captured dynamically over time in transgenic animals subcutaneously injected with 10 μg each of Estradiol (top), and Raloxifene (bottom) while continuously infusing the substrate D-Luciferin intravenously.

**Movie 2**. Video showing the real-time luminescence signal captured dynamically over time in transgenic animals subcutaneously injected with 10 μg each of Diethylstilbestrol (top), Estradiol (middle), and 4-hydroxytamoxifen (bottom) while continuously infusing the substrate D-Luciferin intravenously. Since the transgenic animals were developed with mutant estrogen receptor with no affinity for estradiol, the site injected with estradiol shows no luminescence signal.

**Movie 3**. Video showing the real-time luminescence signal captured dynamically over time in transgenic animals subcutaneously injected with 10 μg each of Diethylstilbestrol (top), Estradiol (middle), and Raloxifene (bottom) while continuously infusing the substrate D-Luciferin intravenously. The signal appears on both sides of abdomen is background signal appears during images set at lower scale. Since the transgenic animals were developed with mutant estrogen receptor with no affinity for estradiol, the site injected with estradiol shows no luminescence signal.

**Movie 4**. Video showing the neoplastic growth formed in female transgenic animals in response to long-term treatment (six months) of BPA treatment. Video showing clear tumor in the flank of animal while the animal is moving.
